# Single cell RNA sequencing research in maternal fetal interface

**DOI:** 10.3389/fcell.2022.1079961

**Published:** 2023-01-10

**Authors:** Qian Chen, Dan Shan, Yupei Xie, Xingrong Luo, Yuxia Wu, Qiuhe Chen, Ruihong Dong, Yayi Hu

**Affiliations:** ^1^ Department of Gynecology and Obstetrics, West China Second University Hospital, Sichuan University, Chengdu, China; ^2^ Key Laboratory of Birth Defects and Related Diseases of Women and Children, Sichuan University, Ministry of Education, Chengdu, China; ^3^ Qingbaijiang Maternal and Child Health Hospital, Chengdu, China

**Keywords:** maternal-fetal interface, single-cell RNA sequencing, trophoblast cell, decidual stromal cells, NK cells, macrophages, T cells

## Abstract

The maternal-fetal interface is an essential environment for embryonic growth and development, and a successful pregnancy depends on the dynamic balance of the microenvironment at the maternal-fetal interface. Single-cell sequencing, which unlike bulk sequencing that provides averaged data, is a robust method for interpreting the cellular and molecular landscape at single-cell resolution. With the support of single-cell sequencing, the issue of maternal-fetal interface heterogeneity during pregnancy has been more deeply elaborated and understood, which is important for a deeper understanding of physiological and pathological pregnancy. In this paper, we analyze the recent studies of single-cell transcriptomics in the maternal-fetal interface, and provide new directions for understanding and treating various pathological pregnancies.

## Introduction

The maternal-fetal interface consists of maternal-derived decidua and fetal-derived placenta (Ander et al., 2019), and includes embryonic-derived trophoblast cells, maternal-derived decidual stromal cells, and decidual immune cells (Yang et al., 2019). Trophoblast cells carry human lymphocyte antigen (HLA) inherited from the paternal lineage, which invade the maternal decidua to anchor the blastocyst to the meconium and participate in the formation of the placenta (Ander et al., 2019). Decidual stromal cells synthesize components of extracellular matrix components, hormones, peptides, cytokines, growth factors, etc., and accumulate glycogen, lipids and proteins to support embryonic growth in early pregnancy (Dey et al., 2004). The decidual immune cells include natural killer (NK) cells, macrophages, dendritic cells, T cells, innate lymphocytes and B cells (Faas and de Vos, 2017; Liu et al., 2017; Vazquez et al., 2019), and play a key role in the immune microenvironment at the maternal-fetal interface and in the establishment of maternal-fetal immune tolerance (PrabhuDas et al., 2015; Liu et al., 2017; Ander et al., 2019). The fact that the fetus acts as a homozygous semi-allograft yet is not rejected by the maternal immune system suggests that complex immune regulatory mechanisms exist between mother and fetus during gestation to maintain homeostasis among the various cell populations as well as within the cells. The imbalance between these cells may lead to pathological pregnancies such as RSA, preeclampsia, preterm delivery and congenital infection (Hoo et al., 2020; Yuan et al., 2020; van 't Hof et al., 2021; Wang et al., 2021b). However, the interactions between cells at the maternal-fetal interface and the dynamics of immune cells are not yet fully described.

Single-cell sequencing is an emerging technology based on the level of individual cell isolation to explore the dynamic differentiation among cell populations and within cells, mainly including Single-cell genomics, transcriptomics, proteomics, epigenomics and interactomics sequencing (Lei et al., 2021). Unlike previous high-throughput sequencing technologies, single-cell genomics not only provides in-depth analysis of individual cell subpopulations measured and the variability of gene expression levels, but also helps to further explore the relationship between cell lineages and cell differentiation trajectories, and to construct the interplay network among cell subpopulations. Currently, research related to single-cell sequencing has gained breakthroughs in several disciplines, including tumor cell heterogeneity, embryonic development, neural activity, immune cell population detection, and plant growth and development (Baslan and Hicks, 2017; Zhao et al., 2018a; Potter, 2018; Shulse et al., 2019; Armand et al., 2021). With the growing development of single-cell sequencing technology, single-cell RNA sequencing (scRNA-seq) has been increasingly applied in the study of pregnancy, and the analysis of various cell types, cell subpopulations and factors such as the variability of gene expression in maternal-fetal interface tissues at the individual cell level has helped to reveal the mechanisms of inter- and intracellular interactive dialogues and thus to better understand cell heterogeneity. This not only promotes the study of cell differentiation and development and cell fate at the maternal-fetal interface, but also advances the research of maternal-fetal medicine. By analyzing the recent studies related to scRNA-seq in maternal-fetal interface heterogeneity in pregnancy, this review aims to deeply explore the heterogeneity of maternal-fetal interface tissues and further understand the potential mechanisms of maternal-fetal immune tolerance to provide guidance direction for the treatment and research of clinical diseases.

### Single-cell RNA sequencing

scRNA-seq, based on the principle that trace amounts of mRNA in isolated individual cells are efficiently amplified and then sequenced at high throughput, is a new technique for high-throughput sequencing of the cellular transcriptome at the level of individual cells. The general procedures of scRNA-seq include the isolation of a single cell, RNA extraction, reverse transcription, preamplification and detection (Kolodziejczyk et al., 2015). In contrast to conventional sequencing, scRNA-seq requires that cell populations in tissues or body fluids are first separated into individual cells, which is precisely where the difficulty lies. The isolation of viable, individual cells from fresh tissues is clearly the most crucial, which determine the accuracy and amount of the amplified dissociation (Lei et al., 2021). The main techniques include micromanipulation, limiting dilution, laser capture microdissection (LCM), fluorescence-activated cell sorting (FACS), magnetic associated cell separation (MACS), immunomagnetic separation and microfluidics (Li et al., 2020; Yasen et al., 2020).

The core strategies include two types: one is to isolate individual cells and construct sequencing libraries independently; the other is labeling (like Barcode) based single cell identification. With strategy one, single cells are isolated one by one and then sequenced separately, which is not only limited by cost but also very low throughput in the face of tens of thousands of cells in one tissue sample. To overcome this limitation, the clever application of droplet-based microfluidics, such as Drop-sep and 10x Genomics has improved the efficiency of single cell identification (Macosko et al., 2015; Zheng et al., 2017): instead of isolating and extracting individual cells one by one, each cell can be tagged with a unique nucleic acid sequence and then sequenced and analyzed. This principle is applied in microfluidics to improve cell throughput with fast cycle times, low cost, and much higher cell capture efficiency.

Methods for scRNA-seq differ in how they tag transcripts for their cell-of-origin and generate libraries for sequencing (Ding et al., 2020), which include Cell expression by linear amplification and sequencing (CEL-seq) (Hashimshony et al., 2012), Smart-seq (Ramskold et al., 2012), Smart-seq2 (Picelli et al., 2014), Drop-seq (Macosko et al., 2015), inDrop-sep (Klein et al., 2015), massively parallel RNA single-cell sequencing (MARS-seq) (Jaitin et al., 2014), 10x Chromium (Zheng et al., 2017), Seq-Well (Gierahn et al., 2017), single-cell combinatorial indexiong RNA sequencing (sci-RNA-seq) (Cao et al., 2017) and so on. Ding (Ding et al., 2020) compared seven methods for single-cell sequencing, including two low-throughput plate-based methods (Smart-seq and CEL-Seq2) and five high-throughput methods (10x Chromium, Drop-seq, Seq-Well, inDrops, and sci-RNA-seq), and found that for the low-throughput methods, Smart-seq2 and CEL-Seq2 performed similarly. Among the high-throughput methods, 10x Chromium was the best-performing method (Ding et al., 2020). Currently, scRNA-seq is widely used to analyze the transcriptome of individual cells at the maternal-fetal interface, while the droplet-based 10X Genomics Chromium is the commonly used platform. As presented in Table 1.

**TABLE 1:** Summary of current scRNA-seq studies on maternal-fetal interface.

Measurement	year	Samples	Gestational age (weeks)	Single cell isolation	Platform	Sequencing	Analysis	Research direction	References
scRNA-seq	2018	Placentas, maternal peripheral blood mononuclear cells and decidua	6–14	enzyme	10X Genomics and Smart-seq2	Illumina Hiseq 2000/4000	Cell Ranger (ver.2.0), HISAT2, R package Seurat (ver.2.3.3)	First trimester	Vento-Tormo et al. (2018)
scRNA-seq	2021	Decidua	39–40	enzyme	10X Genomics	Illumina NovaSeq 6000	Cell Ranger (ver.2.0.1), package of R software, Cell Browser (ver.2.0.0)	Peripartum period	Huang et al. (2021)
scRNA-seq	2019	Basal plate, placental villous, and chorioamniotic membranes	Preterm labor (33–35 weeks) and term labor (38–40 weeks)	MACS, enzyme	10X Genomics	Illumina Hiseq X Ten	Cell Ranger (ver.2.1.1), R package Seurat	Term and preterm parturition	Pique-Regi et al. (2019)
scRNA-seq	2021	placenta	38–41	enzyme	10X Genomics	Illumina NovaSeq 6000	Cell Ranger (ver.3.0), R package Seurat (ver.2.3.4)	Gestational diabetes mellitus	Yang et al. (2021)
scRNA-seq	2018	Villi and decidua	6–11	enzyme	Drop-seq and 10X Genomics	Illumina NextSeq 500 and Illumina HiSeq 2500	Drop-seq core computational protocol (ver. 1.2)	First trimester	Suryawanshi et al. (2018)
scRNA-seq	2018	placenta	8, 24	Enzyme, MACS	Smart-seq2	Illumina Hiseq4000	R package Seurat	first- and second-trimester human placentas	Liu et al. (2018)
scRNA-seq	2022	placenta	17–23	Microscope, enzyme	Smart-seq	Illumina Nova seq 6000	CLC Genomics Workbench 20.0, R (ver. 3.6.1)	Mid-gestation	Toothaker et al. (2022)
scRNA-seq	2021	decidua	6–9	Enzyme, MACS	10X Genomics	Illumina Nova seq 6000	Cell ranger (ver. 3.1.1) and R (ver. 3.5.2)	Unexplained Recurrent pregnancy loss	Chen et al. (2021)
scRNA-seq	2021	decidua	5–8	enzyme	10X Genomics	Illumina Hiseq X Ten	Cell Ranger (ver. 4.0.0 and Seurat (ver. 3.0)R toolkit	Recurrent pregnancy loss	Du et al. (2021)
scRNA-seq	2021	Peripheral blood and decidua	6–8	Enzyme, MACS	10X Genomics	Illumina Nova seq 6000	Cell ranger (ver. 2.0.1), Seurat (ver. 3.0.3) and R package (ver. 3.12.0)	Recurrent pregnancy loss	Wang et al. (2021a)
scRNA-seq	2021	decidua	7–9	Enzyme, FACS	10X Genomics	Illumina Hiseq X Ten	Cell ranger (ver. 3.0.0), Seurat (ver. 2.3.1)	Recurrent pregnancy loss	Guo et al. (2021)
scRNA-seq	2021	placenta	34–39	enzyme	scFTD-seq	Illumina Hiseq X	Ensemble (ver. 92), R package (ver.3.0.1)	preeclampsia	Zhang et al. (2021)
scRNA-seq	2022	placenta	32–41	enzyme	10X Genomics	Not mentioned	Cell ranger, R package Seurat (ver.3.0), SCENIC (ver.1.1.2.2)	preeclampsia	Zhou et al. (2022a)

The 10X genomics Chromium platform builds on the Gem Code technology, which has been frequently used for Single cell transcriptome research in human placenta and decidua (Vento-Tormo et al., 2018; Wang et al., 2021a; Du et al., 2021; Huang et al., 2021; Yang et al., 2021; Zhou et al., 2022a). The core of the technology is a Gel bead in Emulsion (GEM) (Zheng et al., 2017). Each gel bead if functionalized with barcoded oligonucleotides, which are composed of the following parts: (i) sequencing adapters and primers, (ii) a 14 bp barcode drawn from B750,000 designed sequences to index GEMs, (iii) a 10 bp randomer to index molecules (unique molecular identififier, UMI) and (iv) an anchored 30 bp oligo-dT to prime polyadenylated RNA transcripts (Zheng et al., 2017). In the 8-channel microfluidic “double-cross” crossover system of the 10X Genomics Chromium, Gel Beads bind to the cell/enzyme mixture at the first crossover, and then at the second crossover, oil coats the Gel Beads with the cell/enzyme mixture, forming an oil-in-water structure, or GEM (Gel Beads-in-emulsion). After the formation of GEMs, the cells are lysed and the Gel Beads automatically lyse to release a large number of barcode sequences, which are combined with mRNA and subsequently reverse transcribed by mRNA to produce cDNA; the obtained cDNA is purified and enriched for subsequent construction of a standard sequencing library. The cDNA sequences from the same cell carry the same barcode tag specific to the gel microbeads, and each cDNA molecule also carries a specific UMI tag (Kivioja et al., 2011; Zheng et al., 2017; Zheng et al., 2020). In summary, the Gem Code technology enables the separation of individual cells, Barcoding technology distinguishes between different cells and different transcripts of the same gene in the same cell. After the oil droplets are broken, PCR amplification is performed using cDNA with 10× barcode and UMI as a template. Ultimately, mRNA reverse transcription produces cDNA with 10× barcode and UMI information to construct a standard sequencing library (Kivioja et al., 2011; Zheng et al., 2020).

These sequencing libraries are then sequenced on lllumina sequencer and the data is analyzed after sequencing is completed.

### Single-cell RNA sequencing in trophoblast cells

The blastocyst consists of the trophoblast ectoderm, the outer layer of the blastocyst and the inner cell mass (Nakashima et al., 2021), in which the trophoblast proliferates and differentiates into proliferating cytotrophoblast cells (CTBs), which form a cell mass at the tip of the chorion that can differentiate in two directions: the syncytial trophoblasts (STBs) and the extravillous trophoblasts (ETVs) on the fetal side (Saito and Nakashima, 2013). the STB, formed by the fusion of the CTB, is the outer layer of the placental villi that It is in direct contact with maternal glandular secretions and participates in the exchange of nutrients, gases and waste products between maternal and fetal blood, preventing direct contact between maternal and fetal blood (Turco and Moffett, 2019; Nakashima et al., 2021). The STB is the main site of steroid and peptide hormone synthesis and secretion (Burton and Fowden, 2015), and because it does not express MHC class I and II molecules and is not recognized by CD8^+^ T cells, it also acts as a protective immune barrier (Turco and Moffett, 2019).

The invasive effect of EVTs on the metaphase is the initiating and key factor in the formation of the maternal-fetal interface (Pollheimer et al., 2018). Vento-Tormo et al. (2018) use single-cell transcriptomics from first-trimester placentas with matched maternal blood and decidual cells to reveal the upregulation of receptors involved in immunomodulation, cellular adhesion and invasion of EVT. Suryawanshi et al. (2018) performed a scRNA-seq study of early pregnancy placenta and metaphase tissue and found that EVT specifically expresses the extracellular matrix (ECM) glycoprotein *MFAP5*, a component of microfibrils that induces cell motility and cancer cell invasion, demonstrating the invasive and mobile role of EVT (Leung et al., 2014; Suryawanshi et al., 2018). EVT interacts with maternal immune cells and plays an important role in the maternal-fetal tolerance process, such as expressing HLA-C, HLA-E and HLA-G (but not HLA-A and HLA-B) to avoid maternal immune rejection (Moffett and Loke, 2006). Interaction with CD4^+^ T cells leads to an increase in Treg cells and increases the expression of the Treg-specific transcription factor FOXP3 to increase immune tolerance (Tilburgs et al., 2015). Meanwhile, HLA-G expressed by EVT promotes the secretion of large amounts of growth-promoting factors by decidual natural killer cells through the ILT2-KIR2DL4 axis, which promotes fetal growth and development (Fu et al., 2017).

Liu et al. (2018) revealed 14 subtypes of placental cells from first- (8 weeks of gestation) and second-trimester (24 weeks of gestation) human placentas by scRNA-seq. They identified three CTB subtybes (CTB-8W-1, CTB-8W-2 and CTB-8W-3) from the first-trimester placenta. CTB-8W-3 cells highly express cell cycle-related genes, including *RRM2, CCNB1* and *CDK*1, exhibit the highest cell proliferative activity and may act as trophoblast stem cells. CTB-8W-1 has the lowest proliferative capability, but high expression of *Syncytin-2* gene, promotes cell fusion and is the progenitor cell of STB. CTB-8W-2 cell function is not known. EVT in early pregnancy is divided into three subtypes (Liu et al., 2018), which are classified as EVT-8W-1, 2 and 3. EVT-8W-1 highly expresses *RRM2*, has cell proliferative potential and is located in the proximal segment of the cell column, EVT-8W-3 cells are associated with receptor activity regulation and immune response and highly express *Tachykinin-3 (TAC3), fibrinogen activator inhibitor-1 (SERPINE1), PRG2* and *JAM2*, located distal to the cell column. EVT aggregates in mid-gestation to form 2 subtypes, EVT-24W-1 and EVT-24W-2. type 1 is associated with negative regulatory responses to trauma, digestion and the immune system, whereas type 2 is associated with growth regulation, gonadotropin secretion and pregnancy. An ordered pattern of trophoblast differentiation from CTB-8W-2 to CTB-8W-3 and then to EVT-8W and EVT-24W cells was predicted by pseudo-temporal analysis (Liu et al., 2018). The results of Vento-Tormo et al. (2018) et al. also showed that EVT is at the end of the CTB differentiation trajectory. Huang et al. (2021) found that EVT-specific expression of *KRT7, PERP* and *HLA-G* in late pregnancy was divided into five clusters by single-cell transcriptome analysis of metaphase before and after delivery, while there was high heterogeneity in gene expression between each cluster, driving different functions.

Preeclampsia is one of the most serious obstetrical complications, affecting 5%–8% of pregnant women (Hutcheon et al., 2011; Than et al., 2018), and is a principal cause of maternal and perinatal morbidity and mortality (Sarno et al., 2015; Ozimek et al., 2016). Despite the severity of the problem, there is a lack of insight into the molecular pathways that were disturbed earlier (Than et al., 2018). PE is a syndrome caused by multiple factors, mechanisms and pathways, and trophoblast dysfunction in the placenta is a central aspect of PE pathogenesis. A progressive body of evidence supports the conclusion that dysplasia and/or dysfunction of different trophoblast lineages of the placenta play a central role in the pathogenesis of preeclampsia. In the preclinical phase, EVT development may be impaired, resulting in EVT dysfunction, shallow trophoblast invasion, failure of physiological transformation of maternal spiral arteries, abnormal placental blood flow, and histological changes consistent with poor maternal vascular perfusion (Redman and Sargent, 2010; Chaiworapongsa et al., 2014). Zhang et al. (2021) revealed the difference between the preeclampsia and healthy pregnancy groups by analyzing the transcripts of 11,518 cells at the single cell level and found that GO terms enriched in EVT from patients with preeclampsia were mainly associated with immune responses, suggesting altered placental immune function in preeclampsia. Zhou et al. (2022b) classified EVT into four subtypes and found that pro-inflammatory, immune, and oxidative stress-related pathways in preeclampsia EVT subtypes were activated. Further analysis revealed that the transcription factors *CEBPB, ATF3*, and *GTF2B* were significantly reduced in expression in preeclamptic EVT, and *CEBPB* and *GTF2B* were selected for the next functional study, which revealed that knockdown of these two molecules significantly reduced cell viability and invasiveness, and were hypothesized to be involved in the pathogenesis of Pre-eclampsia (Zhou et al., 2022b). Overall, scRNA-seq provides a fresh molecular theoretical basis for preeclampsia trophoblast dysfunction, which may help in the diagnosis and treatment of this disease.

### Single-cell RNA sequencing in decidual stromal cells

Decidual stromal cells (DSC) are a major component of the decidua and secrete a variety of factors that regulate the microenvironment at the maternal-fetal interface in early pregnancy, including IGFBP1, PRL, growth factors, cytokines, and interleukins (Gellersen and Brosens, 2014; James-Allan et al., 2018). Suryawanshi et al. (2018) analyzed the gene expression in more than 20,000 cells from villi and decidua samples by scRNA-seq analysis of human first-trimester (6–11 weeks of gestation) placental and decidual cells using 10x Genomics and Drop-seq platforms. DSC expressed genes involved in *PRL*, *IGFBP1*, *APOA1*, *CHI3L2*, *SEROINA3*, *IL1B*, and *PROk1*. To address stromal cell differentiation, Suryawanshi et al. (2018) placed 1524 stromal cells of decidua in pseudotemporal order and observed the trajectories originating from the fibroblast population toward DSCs. Vento-Tormo et al. (2018) analyzed early gestational (6–14 weeks of gestation) DSC by scRNA-seq and classified them into three cell subpopulations (labeled DS1, DS2, and DS3). The DS1 cell subpopulation expressed *ACTA2* and *TAGLN*, and lacked expression of the classical molecular markers of metaphase, *IGFBP1* and *PRL*. the DS2 and DS3 cell subpopulations expressed *IGFBP1*, *IGFBP2*, and *IGFBP6*, and the DS3 cell subpopulation expressed *PRL* and genes involved in steroid biosynthesis at different locations. They used immunohistochemistry and multiplexed single molecule fluorescent *in situ* hybridization for selected markers on serial section of decidua parietails to demonstrate that DS1 cells are present between glands in the decidua spongiosa, while DS2 and DS3 cells are located in decidua compacta. In addition, they developed a repository of ligand-receptor interacting pairs called CellPhone to identify interactions between decidual NK cells and invading fetal EVTs, maternal immunity, and stromal cells. Using this tool, they predicted that DS2 and DS3 express high levels of *LGALS9* and *CLEC2D*, which could interact with their receptors *TIM3* and *KLRB1*-both expressed by subsets of dNKs-enabling the stroma to suppress the inflammatory response in the decidua (Vento-Tormo et al., 2018). Huang et al. (2021) investigated the transcriptome of 29231 decidual cells before and after delivery (39–40 weeks of gestation) by scRNA-seq and found that the main function of DSC at peripartum period included extracellular matrix organization, protein processing and cell-substrate adhesion.

Recurrent spontaneous abortion (RSA), which is to be defined as the loss of two or more consecutive pregnancies, affects up to 5% of women who are attempting to conceive (Practice Committee of the American Society for Reproductive Medicine. Electronic address, 2020). The known etiologies of RPL include genetic abnormalities, endocrine disorders, uterine malformations, Vitamin D deficiency and other influencing factors such as thrombosis and maternal infections (Garrido-Gimenez and Alijotas-Reig, 2015; Tamblyn et al., 2022). Up to 50% of cases the etiology is not known (Pillarisetty and Mahdy, 2022). Du et al. (2021) analysed a total of 66,078 single cells from decidua samples isolated from patients with RSA (5–8 weeks of gestation) and healthy controls (5–8 weeks of gestation) by scRNA-seq and revealed that RSA samples were accompanied by abnormal decidualization and marked impairment of communication between stromal cells and other cell types, such as abnormal activation of macrophages and NK cells. They identified five clusters of DSCs (DS1-DS5) according to their gene expression profile and found a significant reduction in the number of DSC in RSA decidua and that the genes upregulated in RSA DSC were mainly associated with cellular senescence, wire mesh, apoptosis, endocytosis and autophagy. Meanwhile, a new cell population DS5 appeared in RSA decidua, and DS5 highly expressed genes related to apoptosis and senescence pathways, indicating that DSC underwent abnormal development in RSA decidua. This study confirmed new molecular and cellular mechanisms associated with RSA development.

### Single-cell RNA sequencing in NK cells

Most studies show that the proportion of NK cells in the endometrium of non-pregnant women is low during the follicular and early secretory phases and gradually increases after ovulation (Lee et al., 2010; Drury et al., 2018). NK cells reach their highest proportion in early gestation and are the most abundant leukocytes in maternal-fetal immunity, accounting for about 70% of metaphase leukocytes (Bulmer et al., 1991), and then their number gradually decreases (Williams et al., 2009; Bulmer et al., 2010). The typical phenotype of NK cells is CD3^−^CD56^+^, which can differentiate into different phenotypes in peripheral blood, where about 90% of peripheral blood NK cells (pNK) have a phenotype of CD56^dim^CD16^+^ and can mediate natural and antibody-dependent killing and exhibit high cytotoxicity, while the other 10% of NK cells have a CD56^bright^ CD16-phenotype, produce a variety of cytokines and are less cytotoxic (Koopman et al., 2003; De Maria et al., 2011; Co et al., 2013; Faas and de Vos, 2017; Le Bouteiller and Bensussan, 2017). Decidual NK cells (dNK) have a similar phenotype to adult peripheral blood CD56^bright^CD16^−^CD3^−^. On the other hand, like the CD56^dim^CD16^+^ pNK, dNK cells are rich in granules, including granzyme/granulysin and perforin, but their cytotoxicity is rare and they have a weak ability to kill target cells (Koopman et al., 2003; Le Bouteiller and Bensussan, 2017). dNK cells play an important role in trophoblast invasion, uterine spiral artery remodeling and placenta formation, and promote embryonic development, and maintain maternal tolerance to the embryo (Yang et al., 2019). However, dNK cell imbalance can lead to a variety of adverse pregnancy outcomes, such as recurrent miscarriage, preeclampsia, intrauterine growth restriction, preterm delivery, and intrauterine infection (Zhao et al., 2018b; Faas and De Vos, 2018; Jabrane-Ferrat, 2019).

Vento-Tormo et al. (2018) identified three major dNK populations, namely dNK1, dNK2, and dNK3, which have distinct molecular characteristics and functions. dNK1 cells expressed *CD39, CYP26A1*, and *B4GALNT1*. dNK2 cells expressed *ANXA1* and *ITGB2*, and dNK3 markers expressed *ITGB2*, *CD160*, *KLRB1* and *CD103*. Further analysis revealed that dNK1 cells KIR genes (*KIR2DS1, KIR2DS4, KIR2DL1, KIR2DL2* and *KIR2DL3*), and cytoplasmic granule proteins (*PREF1, GNLY, GZMA* and *GZMB*) were highly expressed and glycolytic metabolism was active. When KIR2DL on dNK cells interacted with HLA-G on EVT, dNK cell activity was inhibited (Tilburgs et al., 2015). It is hypothesized that dNK1 cells interact specifically with EVT to regulate maternal uterine spiral artery remodeling to ensure adequate blood supply for fetal development (Hiby et al., 2004; Li et al., 2009; Hiby et al., 2010; Hiby et al., 2014). dNK3 is highly expressed in *CCL5*, an inflammatory mediator produced by immune cells that has a role in regulating migration and invasion (Yu-Ju Wu et al., 2020), and EVT expresses *CCR1*, a receptor for *CCL5*, suggesting a role for dNK3 in regulating EVT invasion (Vento-Tormo et al., 2018).

Studies suggest that abnormalities in the metaplastic immune microenvironment may be associated with the development of RSA (Triggianese et al., 2016). However, the underlying mechanisms by which dysregulation of the meconium immune microenvironment leads to RSA are not known. Guo et al. (2021) analyzed a total of 18,646 CD45^+^ single transcriptomes from 24 human first-trimester (7–9 weeks of gestation) decidua samples by scRNA-seq. They identified three known subsets of dNK cells (dNK1-3) (Vento-Tormo et al., 2018) and a group of proliferating natural killer cells (dNKp) and found that dNK1 cells express *LILRB1*, which has pro-embryonic growth activity, as well as high expression of angiopoietin receptor Tie-2-mediated signaling pathway genes. dNK2 and dNK3 cells readily secrete cytokines, with dNK3 highly expressing immunomodulatory *IFNG*. Further analysis revealed a significant decrease in dNK1 cells and a significant increase in dNK3 cells in RSA patients. It is hypothesized that in RSA decidua, the presence of dNK has a diminished angiogenic capacity, accompanied by an enhanced pro-inflammatory capacity. Wang et al. (2021a) analyzed a total of 56,758 CD45^+^ single transcriptomes from six human first-trimester (6–8 weeks of gestation) peripheral blood and decidual samples by scRNA-seq and identified five major dNK clusters, named as dNKp, dNK1, dNK2, dNK3, and dNK4. dNKp cells were highly expressed in *MCM5, STMN1*, and *PCNA* and had a significant proliferative capacity. dNK4 cells highly expressed *LILRB1*, a marker gene for memory NK cells, indicating that dNK4 cells are involved in recurrent adverse pregnancy outcomes in recurrent miscarriage. Further comparison of differential dNK cell gene expression between recurrent miscarriage and normal pregnancy revealed that inflammation-related genes were increased in recurrent miscarriage (Wang et al., 2021a). Guo et al. (2021) practical high-resolution pseudo-temporal prediction algorithm Palantir (Setty et al., 2019) postulated that dNKp differentiates into dNK1, dNK2 and dNK3 through three developmental branches, with dNKp differentiation into dNK3 being unique to RSA. It was also determined that dNK2 has a clear tendency to convert to dNK1. Chen et al. (2021) analyzed the transcriptomes of 13,953 CD45+cells from six human first-trimester (6–9 weeks of gestation) decidual samples by scRNA-seq and identified a group of CSF^+^CD59^+^KIRs dNK cells predominantly present in the normal decidua, a subpopulation of cells that performs anti-inflammatory and regulates the function, infiltration and phenotype of multiple immune cells by expressing CD39 and CD59 (Blom, 2017; Zeng et al., 2020). While the proportion of CSF^+^CD59^+^KIRs dNK cells was decreased in RSA (Chen et al., 2021). In summary, the above studies promote our understanding of maternal-fetal interface heterogeneity in disease states from a single-cell perspective and provide new perspectives for clinical immunological studies of RSA.

### Single-cell RNA sequencing in macrophages

Decidual macrophages (dMΦ), phenotypically CD14^+^CD206^+^, account for 20%–30% of maternal immune cells at the site of placental implantation (Heikkinen et al., 2003), constitute the second largest population of metaphase leukocytes in early pregnancy and remain relatively constant in number throughout pregnancy (Vince et al., 1990). dMΦ plays an important role in the establishment and maintenance of normal pregnancy, promoting spiral artery remodeling and trophoblast invasion and maintaining immune tolerance at the maternal-fetal interface by downregulating the inflammatory response and inducing tolerance to embryonic antigens (Petroff et al., 2002; Abrahams et al., 2004; Smith et al., 2009; Faas et al., 2014). Macrophages in tissues are usually divided into two subpopulations: pro-inflammatory M1 and anti-inflammatory M2 cell types (Zhang et al., 2017). dMΦ presents an M1 phenotype before blastocyst implantation, and as pregnancy progresses, after placenta formation and helical artery remodeling, the M2 subpopulation becomes the major macrophage population in the meconium, maintaining maternal-fetal tolerance during pregnancy (Sun et al., 2021).

Yang et al. (2021) analyzed the transcriptomes of 27,220 cells from four freshly placental tissues (two GDM and two normal controls) by scRNA-seq. As presented in Table 1. In their research, 4,306 MΦ were detected, and most of them were M2-polarized. Roger (Pique-Regi et al., 2019) analyzed the transcriptomes of 79,906 cells from three placental compartments: basal plate, placental villous, and chorioamniotic membranes collected from nine women in three study group: term no labor, term in labor, and preterm labor. They found higher *NFKB1* expression in maternal macrophages in women who delivered at term compared to the non-delivery group, and this change was more pronounced in preterm delivery. Differential gene expression in macrophages associated with delivery was mainly involved in the activation of immune responses and regulation of pro-inflammatory cytokine production, suggesting that decidua macrophages undergo M1-type macrophage polarization during full-term and non-full-term deliveries. Wang et al. (2021a) found increased expression of *CXCL8*, *TNF* and *IFIT2* in RSA, while angiogenic factor *VEGFA* and immunosuppressive molecule-encoding gene *LAGLAS1* expression was decreased, presenting a pro-inflammatory state and possibly failing to regulate uterine spiral artery remodeling. Guo et al. (2021) classified dMΦ cells into two cell subtypes, mac1 and mac2, at the single cell level, which were enriched for M1 and M2 genes, respectively, and found that mac1 cells were elevated and mac2 cells were significantly reduced in the RSA, while genes with T Cell tropism were found to be elevated in the expression of dM cellsΦ in the RSA. This once again demonstrated decidual immune microenvironment disorder of patients with RSA.

### Single-cell RNA sequencing in T cells

Decidual T cells are involved in maintaining immune tolerance at the maternal-fetal interface and consist mainly of CD4^+^ T cells, CD8^+^ T cells, and FOXP3+ regulatory T cells (Treg) (Nancy and Erlebacher, 2014). Toothaker et al. (2022) researched the mid-gestation (17–23 weeks of gestation) human placenta villi using single-cell analysis and found that decidua had a higher proportion of CD4T cells compared with either of the fetal layers, and that most of the T cells in placenta villi were of memory phenotypes that could help to limit T cell activation. Chen et al. (2021) revealed that the regulatory role of CD4^+^ T cells and the cytotoxic role of CD8^+^ T cells in early gestation were coordinated to maintain the balance of the decidual immune microenvironment. Guo et al. (2021) studied the heterogeneity of T cells in the recurrent miscarriage and healthy individuals and found enhanced cytokine-mediated signaling pathways and pro-inflammatory properties of various subsets of T cells in the recurrent miscarriage population. It was reported that the normal gestational decidual immune microenvironment is biased toward helper T cells (Th) 2 type, immunity, whereas Th1 immunity may lead to pregnancy failure (Raghupathy et al., 2000), whereas the decidual microenvironment in the recurrent miscarriage population predominantly exhibits Th1 type (Guo et al., 2021). Wang et al. (2021a) similarly confirmed the presence of highly immune activating properties in meconium T cells from RSA patients. Immune factors play an important role in the development of RSA, and correcting the immune imbalance of Th1/Th2 cells may be an important way to reduce the incidence of RSA.

### Single-cell RNA sequencing in dendritic cells

Dendritic cells (DCs) account for about 1%–2% of metaphase free cells and are key antigen presenting cells during pregnancy. DCs can be divided into CD83^+^ mature dendritic cells (mDC), CD209+ (DC-SIGN), immature dendritic cells (imDC) and intermediate state DEC-205 + DCs according to their maturity, with DC-SIGN + DCs predominating in early gestation. Chen et al. (2021) found that most DC cells were in a resting state in early pregnancy, and these resting state dDCs may induce CD8^+^ T cell tolerance in the metaphase microenvironment, while activated state dDCs were found in the recurrent miscarriage population, which may be related to the pathogenesis of RSA.

## Conclusion

Pregnancy is a dynamic and changing process, and the cellular composition in the placenta and decidua may change at each period. In recent years, single-cell genomics technologies have advanced our understanding of tissue heterogeneity at the maternal-fetal interface during pregnancy, advancing the field of maternal-fetal medicine research and providing powerful technical support for studying the physiological and pathological processes of pregnancy. However, we should recognize the limitations of single-cell sequencing. Firstly, single-cell sequencing studies must take into consideration temporal, spatial and individual differences, such as the location of the material, different gestational week, specimen handling procedures and sample size. In addition, single-cell sequencing requires fresh samples and a high percentage of live cells after dissociation, which makes it more difficult to obtain specimens and increases the bias of experimental batches. Finally, scRNA-seq alone cannot link genotype and phenotype, and we still need in-depth multi-omics studies of the maternal-fetal interface supported by single-cell high-resolution technology to ultimately reduce the incidence of maternal-fetal disease during pregnancy and protect maternal-fetal health.
